# Niacin Activates the PI3K/Akt Cascade via PKC- and EGFR-Transactivation-Dependent Pathways through Hydroxyl-Carboxylic Acid Receptor 2

**DOI:** 10.1371/journal.pone.0112310

**Published:** 2014-11-06

**Authors:** Huawang Sun, Guo Li, Wenjuan Zhang, Qi Zhou, Yena Yu, Ying Shi, Stefan Offermanns, Jianxin Lu, Naiming Zhou

**Affiliations:** 1 Zhejiang Provincial Key Laboratory of Medical Genetics, School of Laboratory Medicine and Life Science, Wenzhou Medical University, Wenzhou, Zhejiang, China; 2 College of Life Sciences, Zijingang Campus, Zhejiang University, Hangzhou, Zhejiang, China; 3 Institute of Aging Research, Hangzhou Normal University, Hangzhou, Zhejiang, China; 4 Department of Pharmacology, Max-Planck-Institute for Heart and Lung Research, Bad Nauheim, Germany; University of Pecs Medical School, Hungary

## Abstract

Niacin has been demonstrated to activate a PI3K/Akt signaling cascade to prevent brain damage after stroke and UV-induced skin damage; however, the underlying molecular mechanisms for HCA_2_-induced Akt activation remain to be elucidated. Using CHO-K1 cells stably expressing HCA_2_ and A431 cells, a human epidermoid cell line with high levels of endogenous expression of functional HCA_2_ receptors, we first demonstrated that niacin induced a robust Akt phosphorylation at both Thr^308^ and Ser^473^ in a time-dependent fashion, with a maximal activation at 5 min and a subsequent reduction to baseline by 30 min through HCA_2_, and that the activation was significantly blocked by pertussis toxin. The HCA_2_-mediated activation of Akt was also significantly inhibited by the PKC inhibitors GF109203x and Go6983 in both cell lines, by the PDGFR-selective inhibitor tyrphostin A9 in CHO-HCA_2_ cells and by the MMP inhibitor GM6001 and EGFR-specific inhibitor AG1478 in A431 cells. These results suggest that the PKC pathway and PDGFR/EGFR transactivation pathway play important roles in HCA_2_-mediated Akt activation. Further investigation indicated that PI3K and the G_βγ_ subunit were likely to play an essential role in HCA_2_-induced Akt activation. Moreover, Immunobloting analyses using an antibody that recognizes p70S6K1 phosphorylated at Thr^389^ showed that niacin evoked p70S6K1 activation via the PI3K/Akt pathway. The results of our study provide new insight into the signaling pathways involved in HCA_2_ activation.

## Introduction

Nicotinic acid has long been believed to have a favorable effect on plasma lipids, lowering plasma LDL-cholesterol and raising HDL-cholesterol [1]. Previous clinical data have also demonstrated its beneficial effects in reducing cardiovascular events and mortality in patients with coronary heart disease [2]–[5]. The discovery of G protein-coupled receptor GPR109A (HM74a), recently designated hydroxyl-carboxylic acid receptor 2 (HCA_2_) because the ketone body β-hydroxybutyrate has been identified as its endogenous ligand [6], as a high-affinity receptor for nicotinic acid [7]–[9] has drawn significant attention to the potential development of novel agonists with antilipolytic activity.

HCA_2_ is a G_i_ protein-coupled receptor. Upon activation by niacin, HCA_2_ evokes an inhibitory effect on adenylate cyclase, leading to a decrease in the intracellular cAMP, and meanwhile also elicits a transient rise in the intracellular Ca^2+^ level in a pertussis toxin (PTX)-sensitive manner [7], [8], [10]. In adipocytes, the reduction in intracellular cAMP results in the decreased activity of protein kinase A (PKA), leading to the decreased activity of hormone-sensitive lipase and a reduced triglyceride hydrolysis to free fatty acids [11]. A recent study using LDL-receptor knockout mice lacking the HCA_2_ receptor demonstrated that niacin did not cause a decrease in the plasma free fatty acid level, but retained its effect on the plasma HDL and triglycerides, suggesting that the lipid-modifying properties of niacin are not mediated through HCA_2_ [12]. However, niacin exhibited beneficial effects on the progression of atherosclerosis via HCA_2_ expressed in bone marrow-derived immune cells, but without affecting the plasma lipid profile [13]. Moreover, accumulating evidence convincingly illustrated that niacin mediates its anti-inflammatory effects via HCA_2_-dependent mechanisms in monocytes and macrophages [14], [15], adipose tissue [16], and vascular endothelium [16].

It is well known that extracellular signals transduced by both receptor tyrosine kinases (RTKs) and GPCRs converge upon the activation of a family of phosphoinositide 3-kinases (PI3Ks), followed by the initiation of a phosphorylation cascade leading to the activation of Akt, also known as protein kinase B [17]. The PI3K/Akt signaling pathway plays a major role in the control of cell proliferation, survival, metabolism and nutrient uptake in a cell-type-specific manner through a variety of downstream targets [18], [19]. A growing body of evidence suggests a role for PI3K/Akt signaling in the regulation of the inflammatory response in diseases including rheumatoid arthritis [20], multiple sclerosis [21], asthma [22], and atherosclerosis [23]. Niacin has been shown to exert its protective effects on stroke [24] and UV-induced skin damage [25] via PI3K/Akt-mediated anti-apoptotic pathways. However, the mechanism(s) underlying the regulation of the PI3K/Akt pathway by HCA_2_ is poorly understood.

Our previous data have shown that upon stimulation by niacin, activated HCA_2_ results in the dissociation of G_i_ proteins from G_βγ_-subunit, causing the PKC pathway to couple to ERK1/2 phosphorylation at early time points (≤2 min), and the MMP/EGFR transactivation pathway to act at both early and later time points (2–5 min) [26]. We also present evidence that the βγ-subunit plays a critical role in HCA_2_-activated ERK1/2 phosphorylation. In the present study, we used Chinese hamster ovary (CHO) cells recombinantly expressing human HCA_2_ receptors (CHO-HCA_2_), and A431 cells, a human epidermoid carcinoma cell line that endogenously express functional human HCA_2_ receptors [27], to characterize the regulation of the PI3K/Akt signaling pathway mediated by the human HCA_2_. We found that niacin-mediated activation of human HCA_2_ signals to the PI3K/Akt cascade via the G_i_ protein-initiated PKC and PDGFR/EGFR transactivation-dependent pathways. We also demonstrate that the G_βγ_ subunit plays a key role in the HCA_2_-mediated activation of the PI3K/Akt pathway via interaction with RTK signaling. The results of our study add new understanding to the roles of the HCA_2_ receptor in its beneficial effects on the progression of atherosclerosis.

## Materials and Methods

### Materials

Opti-MEM I reduced serum medium and G418 were purchased from Invitrogen (Carlsbad, CA, USA) and the X-tremeGENE HP reagent was purchased from Roche (Basel, Switzerland). Cell culture medium and fetal bovine serum were obtained from Hyclone (Beijing, China). Alternative Thioglycollate Medium, Pertussis toxin (PTX), GF109203X (bisindolylmaleimide), Go6983, and tyrphostin A9 were obtained from Sigma (St. Louis, MO, USA), while U0126, tyrphostin AG1478, GM6001, PP2 and Wortmannin were from Calbiochem (La Jolla, CA, USA). Anti-phospho-Akt (Ser473), Anti-phospho-Akt(Thr308), Anti–Akt, Anti–EGFR, Anti–PDGFR, Anti-phospho-EGFR (Tyr1173), Anti-phospho-PDGFR (Tyr1018) and the horseradish peroxidase substrate were bought from Cell Signaling Technology (Danvers, MA, USA). Horseradish peroxidase-conjugated goat anti-rabbit secondary antibody and anti-β-actin antibody were obtained from HuaAn Biotechnology (Hangzhou, China). RIPA lysis buffer and a BCA kit were bought from Beyotime (Haimen, China).

### Mice

*Hca_2_* ^+/−^ mice were maintained in specific pathogen-free husbandry. Wild-type and *Hca_2_* ^−/−^ mice were obtained by intercrossing *Hca_2_* ^+/−^ mice. Genotyping of the *Hca_2_* alleles and the inactivated alleles were performed as described [8]. All animal work was conducted in accordance with the Guide for the Care and Use of Laboratory Animals as adopted and promulgated by the United States National Institutes of Health. The protocol was approved by the research ethics committee of Zhejiang University.

### Cell lines and cell culture

CHO-K1 cells (from the American Type Culture Collection) [28] were kindly provided by Dr. Jeffrey Benovic (Thomas Jefferson University, Philadelphia, USA), and were grown in 50∶50 Dulbecco’s modified Eagle’s medium (DMEM)/Ham’s F-12 medium supplemented with 10% fetal bovine serum (FBS) and 2 mM glutamine. A431 cells were obtained from Type Culture Collection of Chinese Academy of Sciences (Shanghai, China) and were cultured in DMEM medium supplemented with 10% FBS and 2 mM glutamine. Cells were maintained at 37°C in a humidified incubator containing 5% CO_2_. Stable cell lines were produced by transfection of CHO-K1 cells with pCDNA3.1-HCA_2_ or pCDNA3.1-HCA_3_ using the X-tremeGENE HP reagent according to the manufacturer’s instructions and selected using G418 [26]. Surviving cells were cloned by limiting dilution, and cell clones were tested for receptor expression by functional analysis using a CRE-driven luciferase activity reporter gene assay. When needed to overexpress a function-deficient protein to detect receptor signaling, 0.6 µg HCA_2_ plasmids plus 2.4 µg Gα-transducin were transiently transfected into CHO-K1 cells or 3 µg βARK1-CT into CHO-HCA_2_ stable cells. pCDNA3.1 was used as a control plasmid.

### Macrophage isolation

4 to 6 weeks old mice were injected with 1 ml 4% Alternative Thioglycollate Medium for three days and macrophages were isolated according to the standard methods [29]. The primary mouse macrophages were maintained in Modified Roswell Park Memorial Institute (RPMI)-1640 medium supplemented with 10% FBS and 2 mM glutamine.

### Immunoblotting assay

CHO-K1 cells or A431 cells were seeded in 24-well plates, rinsed with serum-free DMEM/F-12 or DMEM when grown to 80% confluence and incubated overnight in serum-free medium. After treatment with niacin, the cells were lysed with RIPA buffer. When needed, the cells were preincubated with inhibitors (PTX overnight or other inhibitors for 1 h) prior to treatment with niacin. Total protein was determined using a BCA kit. Equal amounts of total cell lysate were size-fractionated by SDS-PAGE (10–12%) and transferred to a PVDF membrane (Millipore). Membranes were blocked in blocking buffer (TBS containing 0.05–0.1% Tween-20 and 5% nonfat dry milk) for 1 h at room temperature and incubated overnight at 4°C with rabbit monoclonal antibody to Phospho-Akt(Ser^473^), Phospho-Akt(Thr^308^), Phospho-p70S6K1, Phospho-ERK, or β-Actin followed by incubation with an anti-rabbit HRP-conjugated secondary antibody according to the manufacturer’s protocols. The chemiluminescence was detected with a HRP substrate using a film-based system and quantified using the Bio-Rad Quantity One Imaging system (Bio-Rad Laboratories).

### Data analysis

All results are expressed as the mean ± S.E. Data were analyzed using either non-linear curve fitting (GraphPad PRISM version 5.0) or a two-way ANOVA in grouped analysis. Statistical significance was determined using Student’s t test. Probability values less than or equal to 0.05 were considered significant.

## Results

### Niacin induces Akt phosphorylation on both residue Thr^308^ and Ser^473^ through HCA_2_

**Figure 1:**
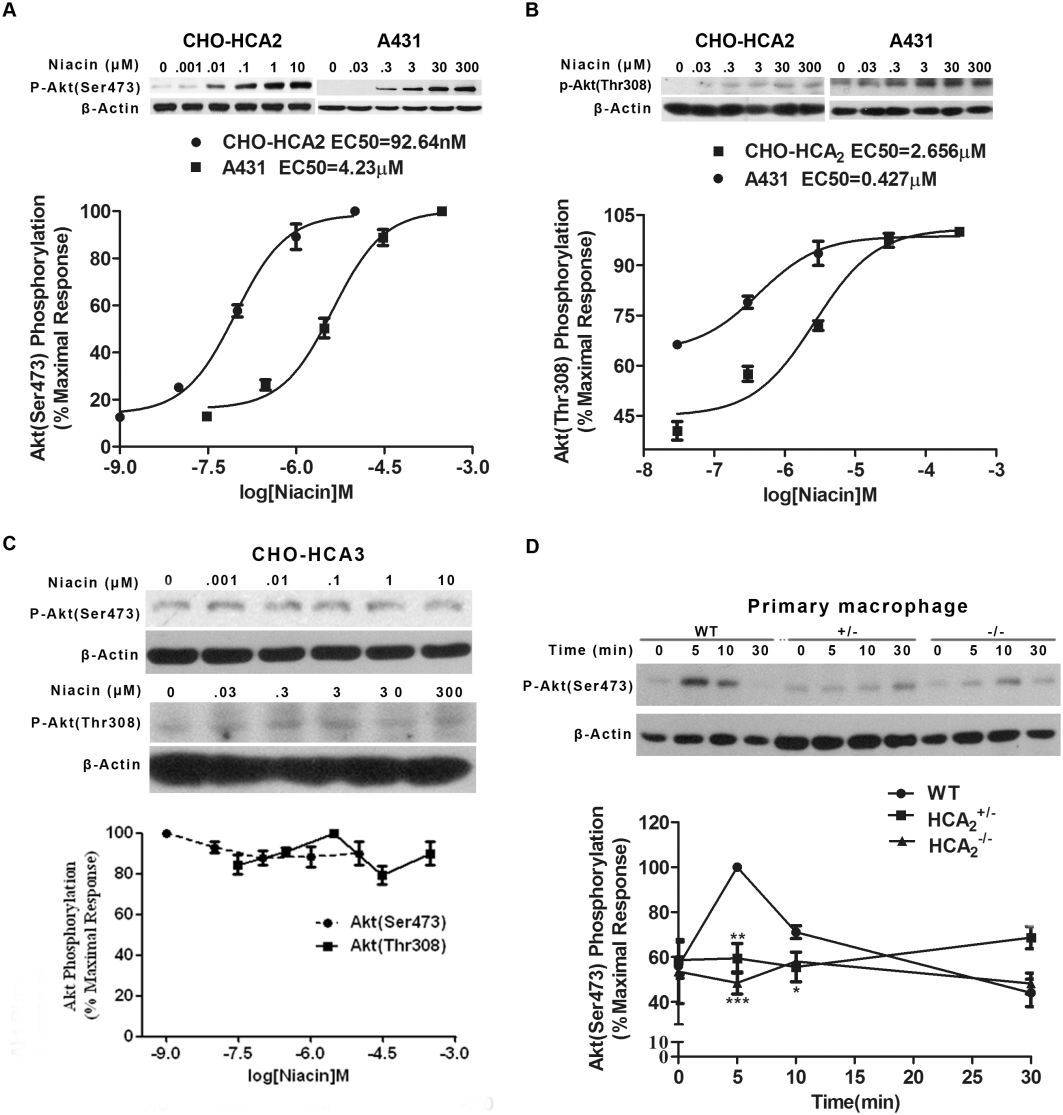
Dynamics of Akt phosphorylation under different concentrations of niacin. CHO-HCA_2_ (A), A431 (B) or CHO-HCA_3_(C) cells were plated on 24-well plates with DMEM/F-12 or DMEM. 12 hours later, the medium was changed to serum-free DMEM/F-12 or DMEM. After overnight starvation, all the cells were treated with different concentrations of niacin for 5 min. Akt phosphorylation at both Ser^473^ and Thr^308^ were detected by Immunobloting. Primary macrophages (D), isolated from 4–6 week old mice, were plated on 24-well plates with modified (RPMI)-1640 medium overnight. Cells were then starved for 3 h and stimulated with 400 µM niacin for different times. Akt phosphorylation at Ser^473^ was detected by Immunobloting. WT: wild type mouse. The data shown are representative of at least three independent experiments. The data were analyzed using Student’s t test (*, p<0.05; **, p<0.01; ***, p<0.001).

Our previous study has demonstrated that niacin induces ERK1/2 activation via PKC- and EGFR-dependent pathways through HCA_2_ in CHO-K1 and A431 cells [26]. In this study, the same CHO-K1 cell line stably expressing the human HCA_2_ was used to determine whether HCA_2_ regulates Akt phosphorylation. As shown in Figs. 1A, 1B, and Fig. S1A, niacin induced robust Akt phosphorylation in both the activation loop within the kinase domain [A-loop (Thr^308^)] and the hydrophobic motif in the C-terminal region [HM (Ser^473^)] in a concentration-dependent manner. Akt phosphorylation in response to niacin was undetectable in CHO-HCA_3_ cells (Fig. 1C), suggesting a specific activation of Akt via HCA_2_ by niacin. Using A431 cells endogenously expressing HCA_2_, niacin-induced Akt phosphorylation on both Thr^308^ and Ser^473^ was observed at comparable levels to that in CHO-HCA_2_ cells (Figs. 1A and 1B, and Fig. S1A and S1B). We next utilized primary macrophages from Alternative Thioglycollate Medium-pretreated HCA_2_-deficient mice or their wild-type littermates to further assess the role of HCA_2_ in niacin-mediated Akt activation. As indicated in Fig. 1D, niacin was found to significantly induce Akt phosphorylation in wild-type macrophages. In contrast, no activation of Akt in HCA_2_-deficient macrophages was detected in the presence of niacin. Taken together, these data suggest that niacin triggers Akt activation through HCA_2_.

### HCA_2_ activates the Akt signaling pathway via a PTX-sensitive G_i_ protein-dependent pathway

**Figure 2:**
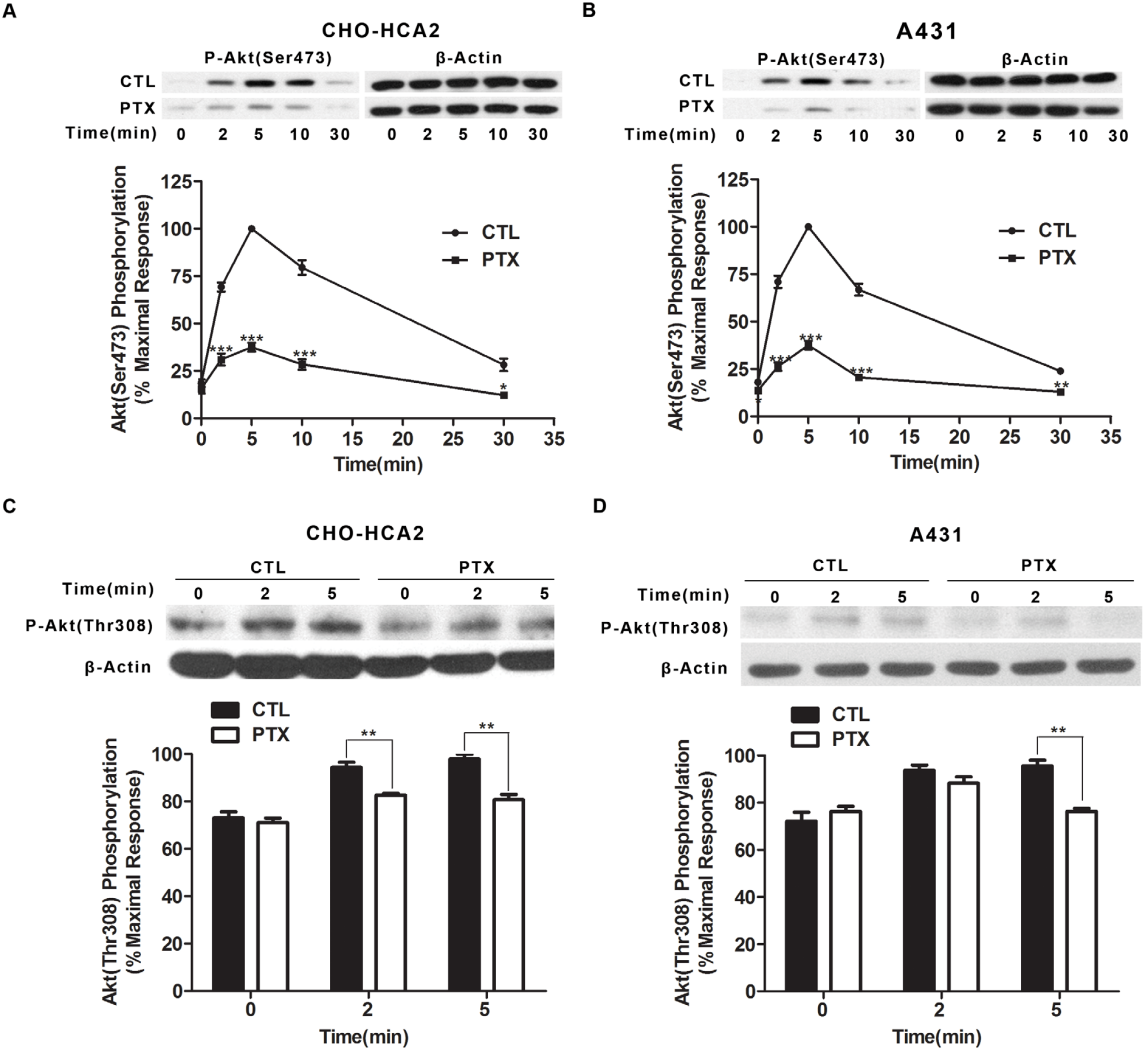
Akt phosphorylation at both Ser^473^ and Thr^308^ was decreased after PTX treatment. Both CHO-HCA_2_ cells (A and C) and A431 cells (B and D) were treated with 100 ng/ml PTX overnight before niacin stimulation (1 µM niacin for CHO-HCA_2_ cells and 100 µM niacin for A431 cells when Akt phosphorylation at Ser^473^ was detected, while 300 µM niacin was used for stimulation for both cell lines when Akt phosphorylation at Thr^308^ was detected) for the indicated time and Akt phosphorylation at Ser^473^ (A and B) and Thr^308^ (C and D) were detected by Immunobloting. The data shown are representative of at least three independent experiments. The data were analyzed using Student’s t test (*, p<0.05; **, p<0.01; ***, p<0.001).

HCA_2_ is associated with G_i_ protein, and upon activation by niacin, acts to inhibit adenylyl cyclase, resulting in the inhibition of forskolin-induced cAMP accumulation. To explore the role of G_i_ protein in the niacin-mediated activation of Akt, CHO-HCA_2_ and A431 cells were cultured in the presence or absence of 100 ng/ml pertussis toxin (PTX) in serum-free medium overnight, followed by stimulation with 1 µM niacin for CHO-HCA_2_ cells and 100 µM niacin for A431 cells. As shown in Fig. 2, niacin evoked significant Akt phosphorylation on both Thr^308^ and Ser^473^ in a time-dependent fashion, with maximal activation at 5 min and with a subsequent reduction to baseline by 30 min. This activation in both CHO-HCA_2_ (Figs. 2A and 2C) and A431 (Figs. 2B and 2D) was remarkably inhibited by pretreatment with PTX, suggesting that HCA_2_ signals through the Akt pathway via a PTX-sensitive G_i_ protein-dependent mechanism.

### Involvement of PKC in HCA_2_-mediated Akt activation

**Figure 3:**
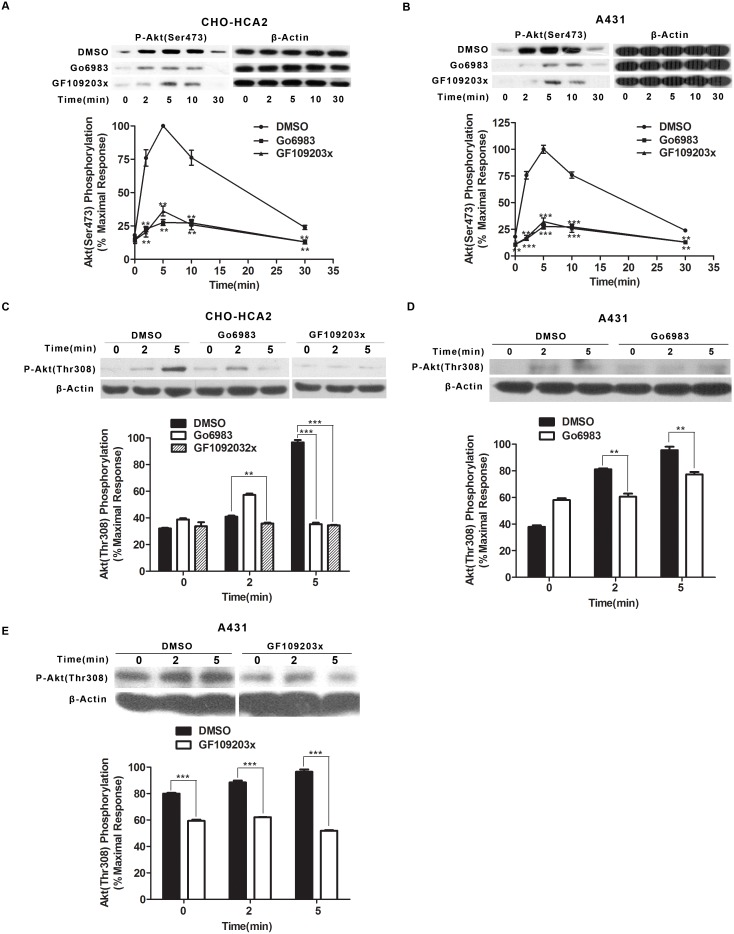
Go6983 and GF109203x decreased Akt phosphorylation at both Ser^473^ and Thr^308^ in CHO-HCA_2_ and A431 cells. Both CHO-HCA_2_ cells (A and C) and A431 cells (B, D and E) were treated with either 10 µM Go6983 or 10 µM GF109203x for 1 h and Akt phosphorylation at Ser^473^ (A and B) and Thr^308^ (C–E) were detected. The data shown are representative of at least three independent experiments. The data were analyzed using Student’s t test (**, p<0.01; ***, p<0.001).

Our previous studies have shown that PKC plays a determinant role in HCA_2_-mediated ERK1/2 activation at early time points (≤2 min) [26]. To investigate whether PKC plays a role in niacin-stimulated Akt phosphorylation via HCA_2_, CHO-HCA_2_ and A431 cells were pretreated with the PKC inhibitors GF109203x (10 µM) or Go6983 (10 µM) for 1 h, followed by niacin stimulation for the indicated time. Both PKC inhibitors exhibited inhibitory effects on niacin-induced Akt phosphorylation at Thr^308^ and Ser^473^ in both CHO-HCA_2_ (Figs. 3A and 3C, and Fig. S2D) and A431 cells (Figs. 3B, 3D, and 3E, Fig. S2C, and S2E). Collectively, these data clearly show that PKC plays a critical role in HCA_2_-mediated Akt activation.

### HCA_2_-induced Akt activation is dependent on a growth factor receptor-involved transactivation mechanism

**Figure 4:**
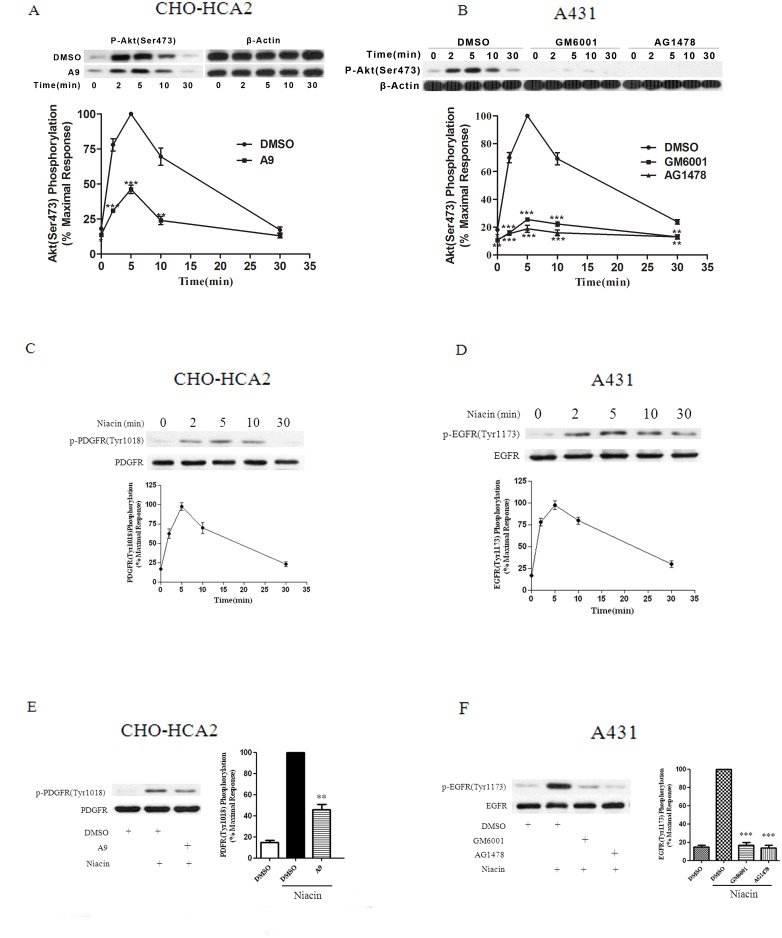
Akt phosphorylation was reduced by A9 treatment in CHO-HCA_2_ cells and by GM6001 and AG1478 treatment in A431 cells. CHO-HCA_2_ cells (A) were treated with 1 µM A9 for 1 h, while A431 cells (B) were treated with 10 µM GM6001 and 100 nM AG1478 for 1 h, then cells were stimulated with 1 µM or 100 µM niacin for indicated time, Akt phosphorylation at Ser^473^ was detected. C and D, Serum-starved CHO-HCA_2_ (C) or A431 (D) cells were stimulated with 1 µM or 100 µM niacin for 5 min, and PDGFR phosphorylation at Tyr^1018^ (C) and EGFR phosphorylation at Tyr^1173^ (D) were detected. E and F, CHO-HCA_2_ cells (E) were treated with 1 µM A9 for 1 h, while A431 cells (F) were treated with 10 µM GM6001 and 100 nM AG1478 for 1 h, then cells were stimulated with 1 µM or 100 µM niacin for 5 min, PDGFR phosphorylation at Tyr^1018^ (E) and EGFR phosphorylation at Tyr^1173^ (F) were detected. The data shown are representative of at least three independent experiments. The data were analyzed using Student’s t test (**, p<0.01; ***, p<0.001).

It is generally accepted that the transactivation of growth factor receptors participates in the GPCR-mediated activation of the ERK/MAPK pathway and phosphorylation of Akt/PKB, induction of cell proliferation and migration [30], [31]. CHO-K1 cells are known to endogenously express PDGF receptor-β but lack EGFR [32]; however, A431 cells have been shown to express EGFR and be devoid of endogenous α- and β-PDGF receptors [33]. CHO-HCA_2_ and A431 cells were preincubated with the PDGF receptor-selective receptor tyrosine kinase inhibitor tyrphostin A9 (1 µM) for 1 h followed by niacin stimulation for different lengths of time. As shown in Fig. 4A, in the tyrphostin A9-pretreated CHO-HCA_2_ cells, there was approximately 60% inhibition of Akt phosphorylation compared with cells treated with agonist alone, whereas there was no inhibition of Akt phosphorylation in the tyrphostin A9-pretreated A431 cells (data not shown). These data demonstrate that PDGFR transactivation is involved in HCA_2_-induced Akt activation in CHO-K1 cells, but not in A431 cells.

To assess the role of EGFR transactivation in niacin-induced Akt activation in cells that endogenously express HCA_2_, A431 cells were utilized for further investigation. Serum-starved A431 cells were treated with AG1478 (100 nM), an EGFR-specific tyrosine kinase inhibitor, for 1 h before exposing them to 100 µM niacin. As shown in Fig. 4B, Fig. S2C, S2D, and S2E, AG1478 dramatically inhibited (>80%) niacin-induced Akt phosphorylation. Several studies have shown that transactivation of EGFR is sensitive to matrix metalloproteinase (MMP) inhibitors [34], [35]. To define the mechanism underlying niacin-induced transactivation of the EGFR, A431 cells were treated with the MMP inhibitor GM6001 (10 µM) for 1 h before niacin stimulation. GM6001 treatment led to a significant reduction (>70%) in Akt activation when induced by niacin (Fig. 4B).

We next examined whether HCA_2_ is able to induce EGFR phosphorylation in A431 cells and PDGFR phosphorylation in CHO-HCA_2_. As shown in Fig. 4C and 4D, niacin stimulated EGFR and PDGFR phosphorylation in a time-dependent manner. Moreover, using specific inhibitors GM6001 and AG1478, EGFR phosphorylation was significantly blocked in A431 cells, and about 50% PDGFR phosphorylation was inhibited in CH0-HCA_2_ cells by pretreatment with A9. These results demonstrate that HCA_2_ evokes Akt activation via the PDGFR transactivation pathway in CHO-HCA_2_ cells and the EGFR transactivation pathway in A431 cells.

### Involvement of PI3K but not Src in HCA_2_-mediated Akt activation

**Figure 5:**
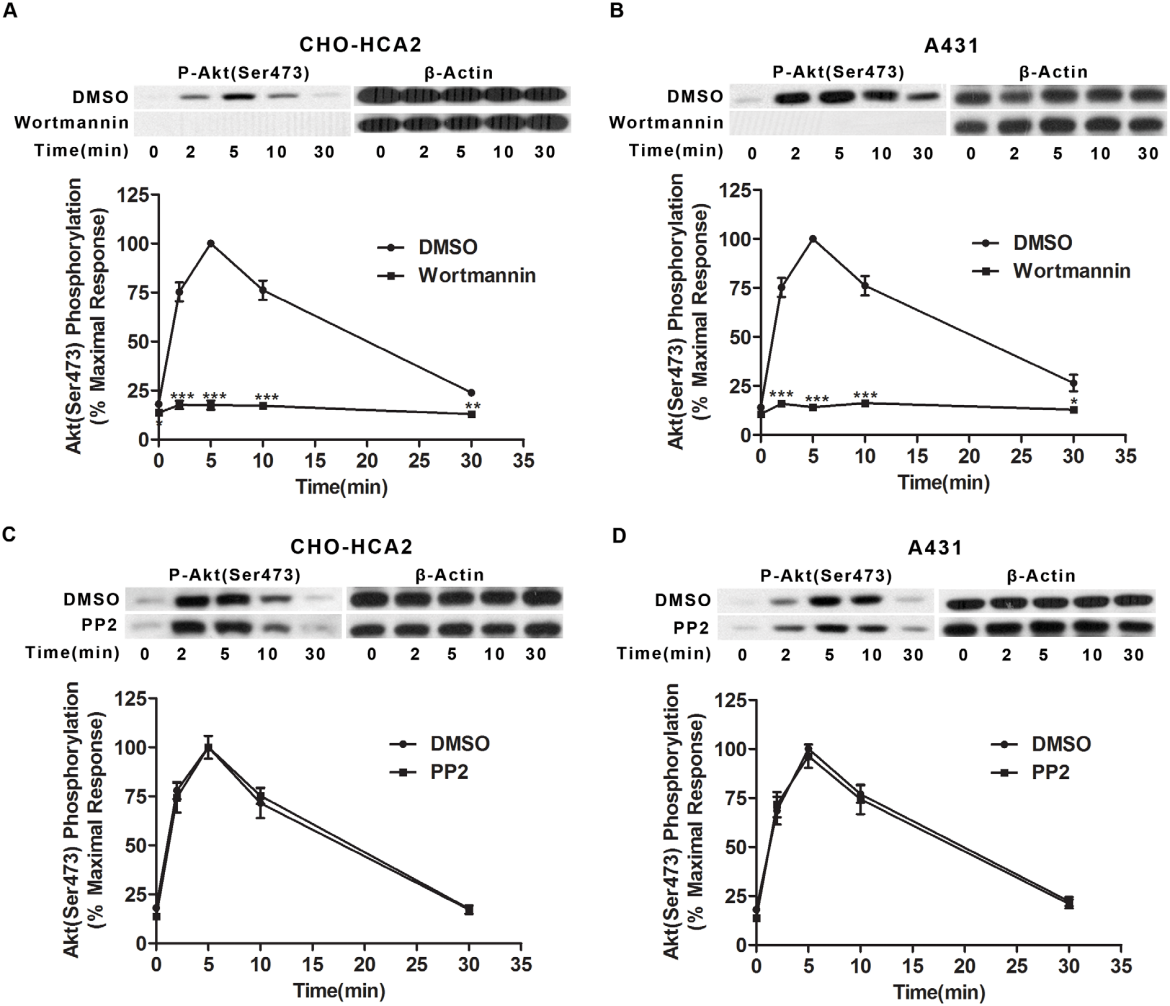
Akt phosphorylation was abolished by Wortmannin treatment, but not by PP2 treatment. Both CHO-HCA_2_ cells (A and C) and A431 cells (B and D) were treated with either 1 µM Wortmannin or 10 µM PP2 for 1 h and Akt phosphorylation at Ser^473^ was detected. The data shown are representative of at least three independent experiments. The data were analyzed using Student’s t test (*, p<0.05; **, p<0.01; ***, p<0.001).

Our previous studies have reported that PI3K and Src are involved in ERK1/2 activation in response to HCA_2_ receptors [26]. Using CHO-HCA_2_ and A431 cells treated with the PI3K inhibitor Wortmannin (1 µM) and the Src inhibitor PP2 (10 µM), we observed that Wortmannin abolished niacin-stimulated Akt phosphorylation in both CHO-HCA_2_ and A431 cells (Figs. 5A and 5B, Fig. S2C, S2D, and S2E), while PP2 had no inhibitory effect on niacin-stimulated Akt activation in either cell line (Figs. 5C and 5D). Collectively, these results show that niacin-induced Akt phosphorylation is PI3K-dependent and Src-independent.

### G_βγ_ plays an essential role in HCA_2_-induced Akt activation

**Figure 6:**
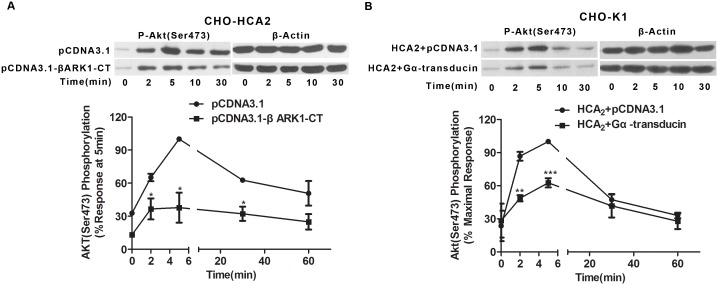
The G_βγ_ subunit involved in HCA_2_ signaling mediates Akt phosphorylation. CHO-HCA_2_ cells (A) were transfected with β-ARK1-CT for 48 h and CHO-K1 cells (B) were co-transfected with Gα-transducin and either pCDNA3.1 or HCA_2_ for 48 h, followed by niacin stimulation and Akt (Ser^473^) phosphorylation detection. The data shown are representative of at least three independent experiments. The data were analyzed using Student’s t test (*, p<0.05; **, p<0.01; ***, p<0.001).

For most G_i_ protein-coupled receptors, signaling from the activated receptor to PI3K/Akt involves the G_βγ_ subunit of heterotrimeric G proteins [36], [37]. Our previous study has demonstrated a critical role for the βγ-subunit in HCA_2_-activated ERK1/2 phosphorylation [26]. Accordingly, we sought to further define the role of the G_βγ_ subunit in HCA_2_-induced Akt activation. β-adrenergic receptor kinase COOH domain (495–689aa) (βARK1-CT) and Gα subunit of transducin, both of which are scavengers of G_βγ_-subunit [38]–[40], were transfected into CHO-HCA_2_ cells and CHO-K1 cells with HCA_2_, respectively. Upon transfection, a significant inhibition in HCA_2_-mediated Akt phosphorylation was observed (Fig. 6A and 6B), suggesting that the G_βγ_ subunit is likely to play a central role in HCA_2_-induced Akt activation. To investigate the role of G_βγ_ and G_i/o_ in the regulation of phosphorylation of EGFR and PDGFR, G_i/o_ inhibitor PTX and G_βγ_ dominant-negative construct Gα-transducin were used. As shown in Fig. S2A and S2B, in both A431 and CHO-HCA2 cells, pretreatment with PTX or transfection with Gα-transducin resulted in a significant inhibition of niacin-induced EGFR or PDGFR phosphorylation. These results demonstrate that HCA_2_-mediated activation of EGFR or PDGFR is both G_i/o_ and G_βγ_-dependent.

Next, we further explored the pathways of HCA_2_-mediated Akt activation in primary macrophage which express lower level of HCA_2_ compared to A431 cells, as shown in Fig. S2C, HCA_2_ caused Akt activation mainly through PKC and EGFR transactivation-dependent pathways, as the same as observed in A431 cells.

### Niacin stimulates Akt-dependent and ERK1/2-independent p70S6K1 activation

**Figure 7:**
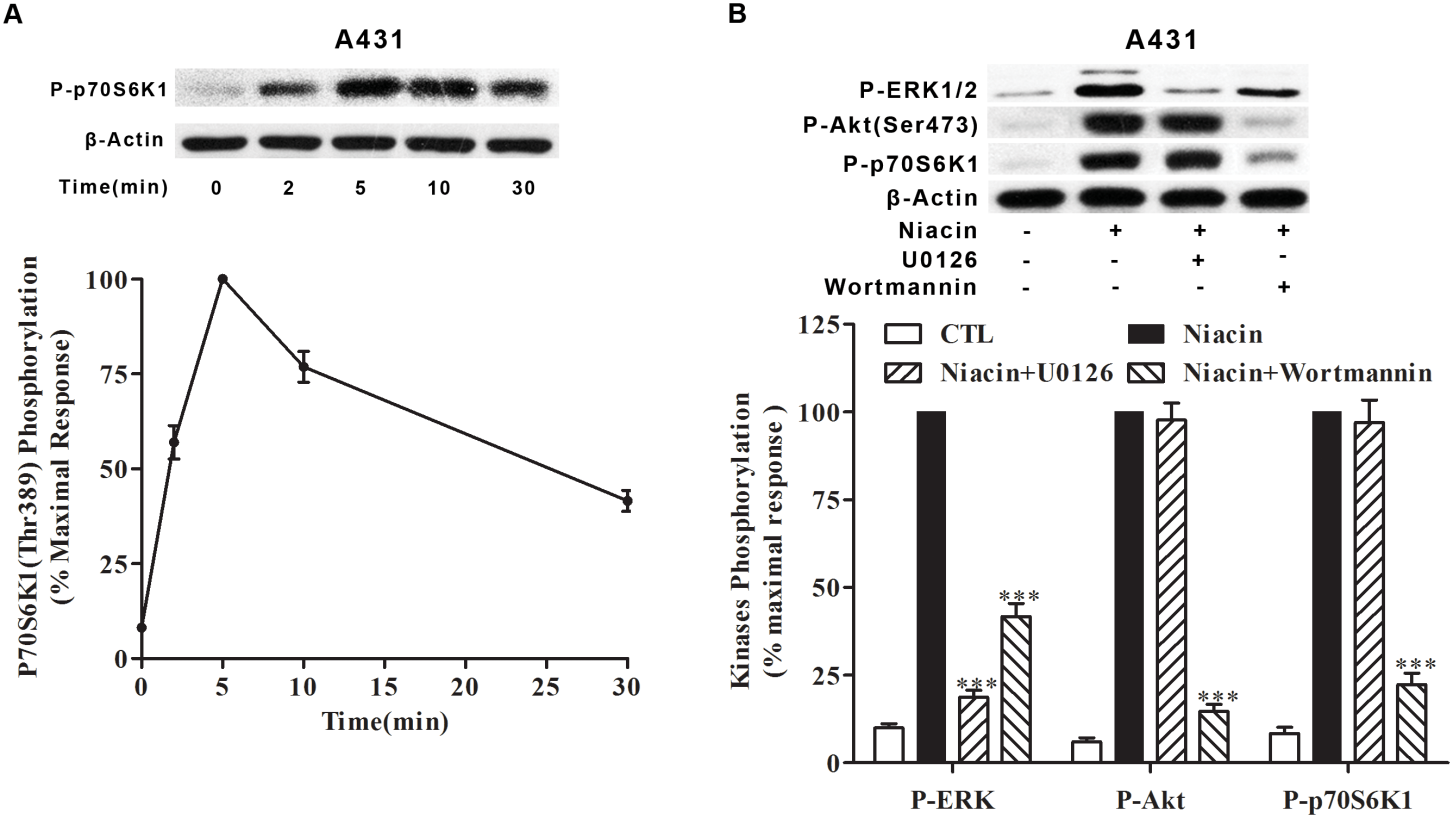
Niacin promotes p70S6K1 phosphorylation through an Akt-dependent but ERK1/2-independent pathway. (A) A431 cells were plated on 24-well plates and were treated with 100 µM niacin for the indicated time after 12 h culture and overnight starvation. p70S6K1 phosphorylation at Thr^389^ was detected by Immunobloting. (B) A431 were treated with either U0126 or Wortmannin with or without niacin stimulation and phosphorylation of Akt (Ser^473^), ERK and p70S6K1 were detected. The data shown are representative of at least three independent experiments. The data were analyzed using Student’s t test (***, p<0.001).

The 70 kDa ribosomal S6 kinase 1 (P70S6K1) is an important regulator for mediating cell growth by inducing protein synthesis and G1 cell cycle progression [41]. Previous studies have reported that P70S6K1 can be activated through the PI3K [42] and MAPK pathways [43]. To determine whether niacin can activate p70S6K1 in A431 cells, a human epidermoid cancer cell, serum-starved A431 cells were stimulated with 100 µM niacin for various times (0–30 min) and lysed, and the extracts were subjected to Immunobloting analyses using an antibody that recognizes p70S6K1 phosphorylated at Thr^389^, a major phosphorylation site that correlates closely with kinase activity [44]. As shown in Fig. 7A, HCA_2_-initiated activation of p70S6K1 occurred in a time-dependent manner, with a maximal activation at 5 min and with a subsequent reduction to 40–50% of the maximal response by 30 min in A431 cells after stimulation with niacin.

To investigate whether HCA_2_-induced p70S6K1 phosphorylation is mediated by ERK1/2 activation or Akt activation, U0126, a highly selective inhibitor of both MEK1 and MEK2, and Wortmannin, a highly selective inhibitor of PI3K, were analyzed for their effect on the activation of p70S6K1. As shown in Fig. 7B, ERK1/2 activation stimulated by niacin was significantly inhibited by preincubation with U0126 (>75%) or Wortmannin (>50%), whereas the Akt phosphorylation stimulated by niacin was only inhibited by preincubation with Wortmannin (>80%), but not U0126. Further, the p70S6K1 phosphorylation mediated by HCA_2_ was also only inhibited by preincubation with Wortmannin (>75%), but not U0126. Taken together, these results demonstrate that HCA_2_ evokes p70S6K1 activation via the PI3K-Akt pathway in A431 cells in response to niacin.

## Discussion

The serine/threonine protein kinase Akt has been shown to play a central role in the regulation of cell survival and proliferation, metabolism, and inflammation in different cell systems through a variety of down-stream effectors [19]. It is generally accepted that Akt, when recruited to the plasma membrane from the cytosol through the binding of its PH domain to the second messenger PIP3 generated by PI3K, is activated by phosphorylation at Thr^308^ in the activation loop and at Ser^473^ within the carboxy-terminus by PDK1 and mTORC2 [19], [45], [46]. Previous studies showed that niacin exerts its protective effects on stroke- [24] and UV-induced skin damage [25] via PI3K/Akt-mediated anti-apoptotic pathways. Therefore, in the present study, to better delineate the signaling pathways linking the HCA_2_ receptor to the PI3K/Akt cascade, we used CHO-K1 cells that were stably or transiently transfected with human HCA_2_ receptors and A431 cells that endogenously express functional human HCA_2_ to characterize HCA_2_-mediated Akt activation through visualization of increases in phosphorylation at both Ser^473^ and Thr^308^ using site-specific antibodies. Our results clearly showed that niacin triggered Akt phosphorylation at both the A-loop (T308) and the HM (S473) in a dose-dependent manner though HCA_2_, leading to the activation of p70S6K1.

The present study determined the roles of various molecular components in the niacin-elicited activation of Akt by HCA_2_ receptors stably or transiently expressed in the CHO-K1 cell line, a cellular model system for investigating GPCR coupling to various signaling pathways. In addition, complementary experiments were performed to further evaluate the effects of niacin in the A431 cell line, a human epidermoid cell line natively expressing functional HCA_2_ [27]. A431 cells have been shown to also express the HCA_3_ receptor, which shares a high degree of similarity with HCA_2_, displaying 96% identity to HCA_2_ but with a 24 amino acid extension at its carboxyl terminus [7], [8], and there are no specific antagonists against HCA_2_ or HCA_3_ available to discriminate between HCA_2_ and HCA_3_ in A431 cells. However, a previous study has revealed that the amount of HCA_2_ mRNA is approximately 1.5-fold more than that of HCA_3_ in A431 cells, supporting the proposition that HCA_2_, rather than HCA_3_, mediates the major effects of niacin on lipolysis [7]. In addition, a recent study has demonstrated that HCA_3_ expressed in CHO-K1 cells failed to evoke Ca^2+^ mobilization in response to stimulation with high concentrations of niacin (up to 1 mM) [47]. Our previous results using concentration curve analysis and siRNA-mediated knockdown of HCA_2_ and HCA_3_ indicated that the role of HCA_3_ in ERK1/2 activation in A431 cells that are stimulated by less than 100 µM of niacin is likely to be negligible or nonexistent [26]. Therefore, it is likely that niacin-induced Akt phosphorylation in A431 cells was mediated by HCA_2_. Moreover, using primary macrophages isolated from Alternative Thioglycollate Medium-treated HCA_2_-KO mice, our data confirmed that niacin triggered Akt phosphorylation through the HCA_2_ receptor.

HCA_2_ is a G_i_ protein-coupled receptor. Upon stimulation by niacin, HCA_2_ inactivates adenylyl cyclase, leading to a decrease in intracellular cAMP levels. Niacin-mediated inhibition of forskolin-evoked cAMP accumulation [7], stimulation of [^35 ^S]GTPγS binding [9], Ca^2+^ mobilization and ERK1/2 activation [8], [26], [48], and anti-lipolytic effects [49] are sensitive to PTX. To determine whether the dominant pathway for HCA_2_-mediated Akt phosphorylation is through G protein activation, we first examined the role of the G_i_ protein in the activation of the Akt signaling cascade. Both CHO-HCA_2_ cells and A431 cells exhibited time-dependent activation of Akt in response to niacin, peaking at approximately 5 min and returning to basal levels at 30 min, but this Akt activation was completely attenuated in the presence of PTX. These results indicate that the heterotrimeric G_i_ protein is essentially involved in the regulation of Akt phosphorylation in both CHO-HCA_2_ and A431 cells. Furthermore, although there is evidence that Akt activation occurs in neural and epithelial cells independently of PI3K [50], it is generally accepted that Akt activation is dependent on PI3K, and inhibition of PI3K activity impairs Akt phosphorylation and Akt-mediated cell functions [19], [45]. Our results showed that HCA_2_-mediated Akt activation was completely blocked in the presence of Wortmannin, a PI3K inhibitor, suggesting that PI3K is an upstream regulator of Akt activation induced by HCA_2_.

The agonist-activated HCA_2_ receptor elicits a rapid increase in intracellular Ca^2+^ in a PTX-sensitive manner [48]. Our previous data have also demonstrated that HCA_2_ couples to ERK1/2 phosphorylation at early time points (≤2 min) via the Go6983 and GF109203x-sensitive PKC-dependent pathway [26]. We thus assess the role of PKC in the regulation of HCA_2_-induced Akt phosphorylation using specific inhibitors. Our data showed that the HCA_2_-elicited Akt phosphorylation was significantly blocked by the broad spectrum PKC inhibitors Go6983 and GF109203x, suggesting that the PKC pathway participates in the activation of Akt, but this activation is distinct from the PKC pathway-mediated ERK1/2 phosphorylation that occurs at early time points (≤2 min) in response to niacin. Previous studies have indicated that both conventional and novel PKC isoforms are found to positively and negatively regulate the activation of Akt [51]–[53]. It is likely for niacin to induce Akt activation via a HCA_2_-mediated PKC-dependent pathway. However, more experiments are necessary to further clarify the exact role of conventional and novel PKC isoforms in the regulation of Akt activation though HCA_2_.

The crosstalk with receptor tyrosine kinases (RTKs), also termed transactivation, has emerged as a common mechanism linking GPCRs to the MAPK and Akt signaling cascades [31], [35]. The role of RTK transactivation is cell-specific; for example, COS-7 cells express the EGF receptor [54], whereas CHO-K1 cells express the PDGF receptor but lack endogenous EGFR [55]. Therefore, experiments using the RTK-selective inhibitors tyrphostin A9 for the PDGF receptor and AG1478 for the EGF receptor were performed to evaluate the role of RTK in the regulation of Akt activation by HCA_2_ in both CHO-HCA_2_ cells and A431 cells. The significant blocking effect of tyrphostin A9 and AG1478 strongly suggested that HCA_2_-mediated Akt phosphorylation required PDGFR-dependent transactivation in CHO-HCA_2_ cells and EGFR-dependent transactivation in A431 cells. Additional data derived from experiments using the MMP inhibitor GM6001 demonstrated that the inhibition of matrix metalloproteinase activity attenuated the HCA_2_-induced activation of Akt, defining the important role of the proteolytic release of heparin-binding EGF-like growth factor (HB-EGF) in the regulation of EGFR transactivation-dependent Akt phosphorylation by HCA_2_ in A431 cells. This is in agreement with our previous evidence that the HCA_2_ receptor induced ERK1/2 activation via a MMP-mediated EGFR transactivation pathway [26]. HB-EGF is synthesized as a membrane-anchored form (pro-HB-EGF) in the cell and is proteolyzed by a metalloproteinase of the zinc-dependent “a disintegrin and metalloproteinase” (ADAM) family to form a soluble growth factor, acting on EGFR as a potent ligand [56], [57]. Different members of the ADAM family, including ADAM10, ADAM12, and ADAM17, mediate GPCR-induced EGFR transactivation in different model systems [58]. The precise mechanism(s) that link GPCRs and their effectors for MMPs activation remain(s) largely unknown. Several kinases, such as Src, PKC and PYK2, were found to regulate MMP activity through direct interaction with MMPs [30]. In the present study, we observed that PKC is involved in the regulation of Akt phosphorylation, whereas the Src kinase is not required for HCA_2_-induced EGFR transactivation in either CHO-HCA_2_ or A431 cells.

In the current study, our results demonstrate that PKC and RTK transactivation are essentially involved in the HCA_2_-mediated PI3K/Akt cascade. This activation is abolished by pretreatment with PTX. In addition, we also observed that overexpression of the G_βγ_ subunit scavenger Gα-transducin effectively attenuated the Akt activation triggered by HCA_2_. This is highly consistent with a model in which G_i_-coupled receptors activate the Akt cascade using G_βγ_-subunit released from G_i/o_ proteins [59]–[61]. There is a growing body of evidence to conclusively suggest that the G_βγ_ subunit from G_i/o_ and G_q_ proteins can directly interact with a selected set of effector molecules, including PLCβ and PI3K [62]. Taken together, our results suggest that activation of the Akt pathway initiated by HCA_2_ is likely to be dependent on G_βγ_-subunit released from G_i_ proteins in a PI3K-dependent manner.

In conclusion, we present evidence that HCA_2_-induced PI3K/Akt activation requires PKC activity and MMP-dependent EGFR transactivation in A431 cells or PDGFR transactivation in CHO-HCA_2_ cells through a mechanism that involves G_βγ_ subunit in a PTX-sensitive manner. However, more research must be performed to fully understand the impact of human HCA_2_ receptor signaling to the PI3K/Akt cascade for niacin in the modulation of atherosclerosis and anti-inflammation.

## Supporting Information

Figure S1 A. Serum-starved CHO-HCA2 and A431 cells were stimulated with 100 µM niacin for 5 min, B. Serum-starved A431 cells were stimulated with various concentrations of niacin for 5 min, cells were harvested, and equal amounts of total cellular lysate were separated by 10% SDS-PAGE, transferred to a PVDF membrane, and incubated with anti-p-Akt(Ser308) antibody. Blots were stripped and reprobed for T-Akt andβ-Actin to control for loading. The data shown are representative of at least three independent experiments.
(TIF)

Figure S2 A and B, CHO-HCA2 cells (A) and A431 cells(B) were treated with 100 ng/ml PTX overnight or transfection of Ga-transducin, then cells were stimulated with 1 µM or 100 µM niacin for 5 min, and PDGFR phosphorylation at Tyr1018 (A) and EGFR phosphorylation at Tyr1173 (B) were detected. Primary macrophage cells (C) and A431 cells (E) were treated with 1 µM wortmannin, 10 µM Go6983, 100 nM AG1478, while CHO-HCA2 cells (D) were treated with 1 µM wortmannin, 10 µM Go6983, 1 µM A9, cells were then stimulated with 1 µM (CHO-HCA2) or 100 µM (A431) or 400 µM (Primary macrophage) niacin for 5 min, and Akt phosphorylation at Ser473 was detected. The data shown are representative of at least three independent experiments. The data were analyzed using Student’s t test (***, p<0.001).
(TIF)
