# Supplementary figures and images for: Niacin Activates the PI3K/Akt Cascade via PKC- and EGFR-Transactivation-Dependent Pathways through Hydroxyl-Carboxylic Acid Receptor 2
Source: PLoS One. 2014 Nov 6;9(11):e112310. doi: 10.1371/journal.pone.0112310 (PMC4223033; doi:10.1371/journal.pone.0112310)

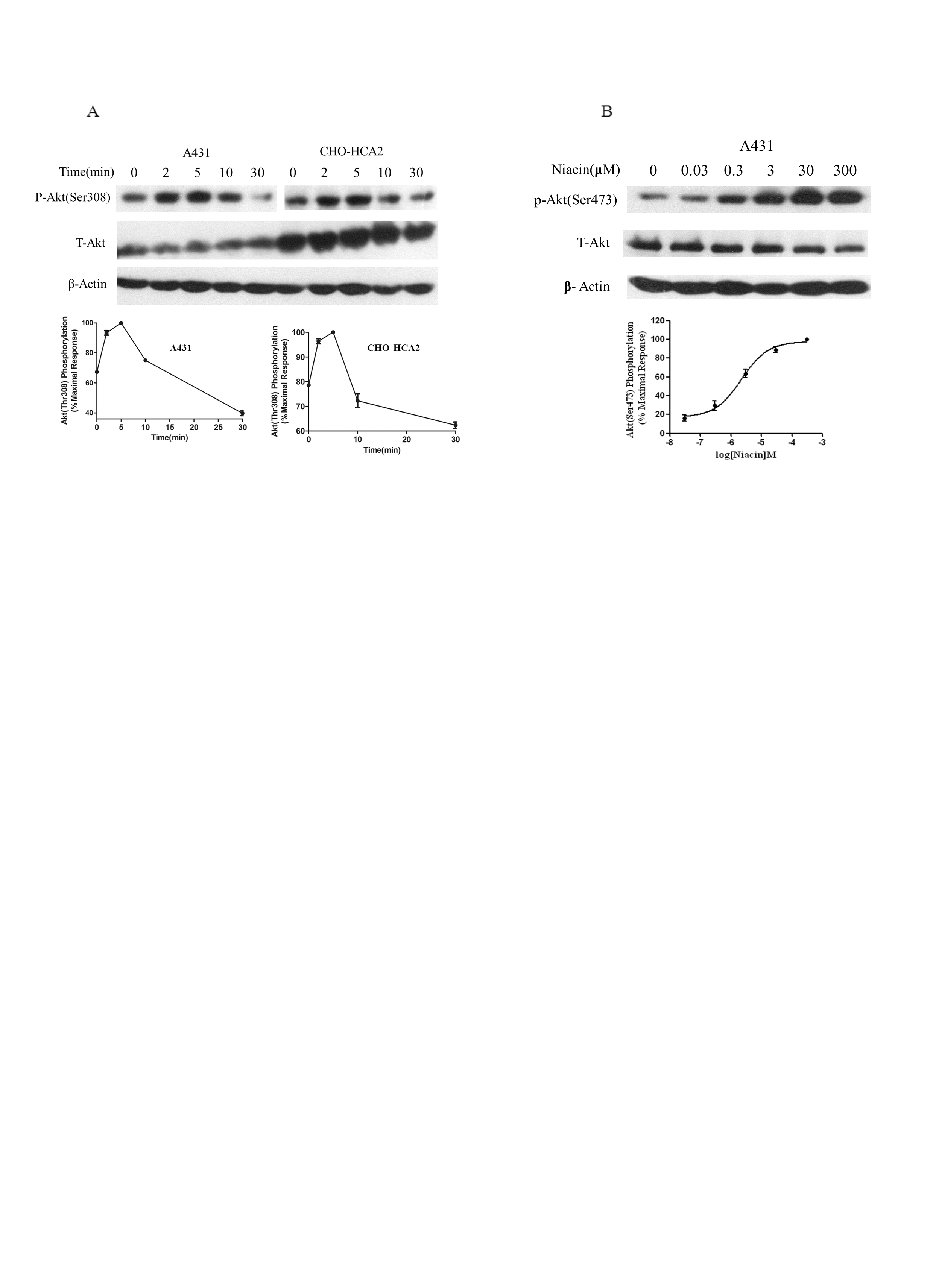

Supplement: Figure S1 — A. Serum-starved CHO-HCA2 and A431 cells were stimulated with 100 µM niacin for 5 min, B. Serum-starved A431 cells were stimulated with various concentrations of niacin for 5 min, cells were harvested, and equal amounts of total cellular lysate were separated by 10% SDS-PAGE, transferred to a PVDF membrane, and incubated with anti-p-Akt(Ser308) antibody. Blots were stripped and reprobed for T-Akt andβ-Actin to control for loading. The data shown are representative of at least three independent experiments. (TIF) [file pone.0112310.s001.tif]

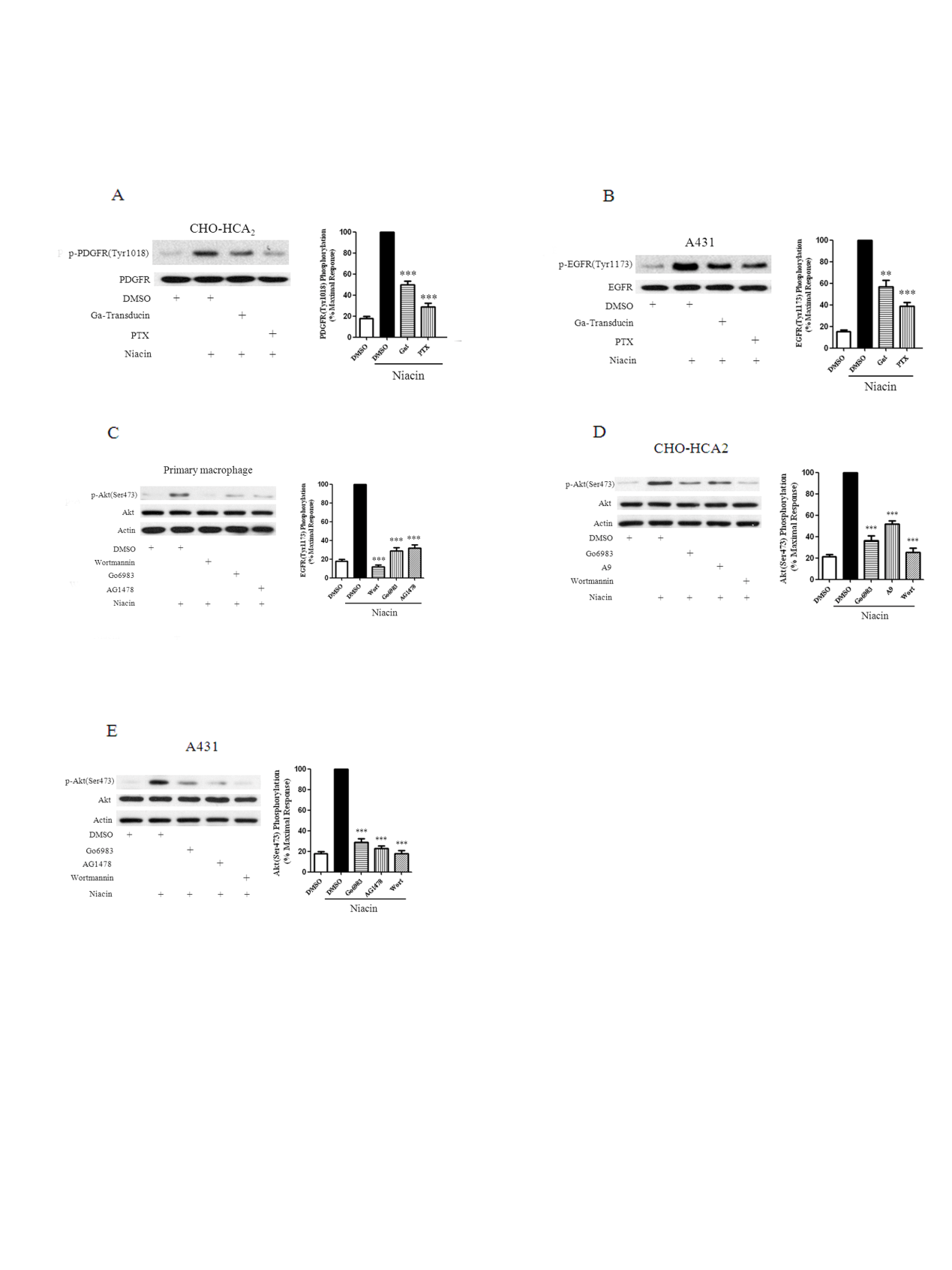

Supplement: Figure S2 — A and B, CHO-HCA2 cells (A) and A431 cells(B) were treated with 100 ng/ml PTX overnight or transfection of Ga-transducin, then cells were stimulated with 1 µM or 100 µM niacin for 5 min, and PDGFR phosphorylation at Tyr1018 (A) and EGFR phosphorylation at Tyr1173 (B) were detected. Primary macrophage cells (C) and A431 cells (E) were treated with 1 µM wortmannin, 10 µM Go6983, 100 nM AG1478, while CHO-HCA2 cells (D) were treated with 1 µM wortmannin, 10 µM Go6983, 1 µM A9, cells were then stimulated with 1 µM (CHO-HCA2) or 100 µM (A431) or 400 µM (Primary macrophage) niacin for 5 min, and Akt phosphorylation at Ser473 was detected. The data shown are representative of at least three independent experiments. The data were analyzed using Student’s t test (***, p<0.001). (TIF) [file pone.0112310.s002.tif]
